# Evidenzbasierte naturheilkundliche Pflegeinterventionen in der Schmerztherapie

**DOI:** 10.1007/s00482-023-00705-w

**Published:** 2023-04-11

**Authors:** Regina Stolz, Elke Kaschdailewitsch, Birgit Kröger, Bettina Noack, Cornelia Mahler

**Affiliations:** 1 Institut für Allgemeinmedizin und Interprofessionelle Versorgung, Universitätsklinikum Tübingen, Tübingen, Deutschland; 2 Abteilung Pflegewissenschaft, Institut für Gesundheitswissenschaften, Universitätsklinikum Tübingen, Hoppe-Seyler-Str. 9, 72076 Tübingen, Deutschland

**Keywords:** Multimodale Schmerztherapie, Akuter Schmerz, Chronischer Schmerz, Wickel und Auflagen, Aromatherapie, Combined modality therapy/pain, Acute pain, Chronic pain, External application, Aromatherapy

## Abstract

Beruflich Pflegende sind in allen Versorgungssettings und bei allen Zielgruppen mit Patient:innen konfrontiert, die an akuten und/oder chronischen Schmerzen leiden. Obwohl in nationalen und internationalen Leitlinien zu chronischen Schmerzen die Bedeutung nichtmedikamentöser Maßnahmen (beispielsweise Wärme- und Kälteanwendungen) und edukativer Ansätze, wie der Vermittlung von Selbstmanagementstrategien im Umgang mit dem Schmerz, betont wird, ist die medikamentöse Therapie die am häufigsten angewendete Maßnahme bei chronischen Schmerzen. Ziel des vorliegenden Beitrags ist es, einen Einblick in das Potenzial naturheilkundlicher Pflegeinterventionen für die Versorgung von Schmerzpatient:innen zu geben, wobei der Fokus auf sogenannten Äußeren Anwendungen wie Wickeln und Auflagen liegt, die risikoarm und einfach anzuwenden sind, sodass sie zur Selbstanwendung geeignet sind. Die Anwendung nichtmedikamentöser Verfahren wie auch die Beratung und Schulung von Patient:innen in den Anwendungen bieten Pflegefachpersonen die Möglichkeit eines autonomen Handlungsfelds und machen den Pflegeberuf attraktiver, da ein eigenständiges und verantwortliches Handeln gefördert wird.

## Abstract (en)

Nurses are confronted with patients suffering from acute and/or chronic pain in all nursing care settings and within all target groups. Although national and international guidelines on chronic pain emphasize the importance of non-pharmacological interventions (for example, hot and cold applications) and educational approaches such as teaching self-management strategies to deal with pain, the most common applied intervention for chronic and acute pain is a pharmacological therapy. The aim of this article is to provide an overview of the potential of naturopathic nursing interventions for the care of patients with chronic and/or acute pain, focusing on so-called external applications such as wraps and compresses, which are safe, have a low-risk profile and are easy to apply, thus making them suitable for self-application. The use of non-pharmacological interventions as well as the counselling and training of patients in naturopathic applications offer nurses the possibility to work in an autonomous field of nursing promoting nursing as an attractive health profession.

## Einordnung der naturheilkundlichen Pflegeinterventionen in den multimodalen Ansatz der Schmerztherapie

Beruflich Pflegende sind in allen Versorgungssettings und bei allen Zielgruppen mit Patient:innen konfrontiert, die an akuten und/oder chronischen Schmerzen leiden. Akuter Schmerz wird dabei definiert als „ein plötzlich auftretender und über einen begrenzten Zeitraum andauernder Schmerz, der durch eine tatsächliche oder drohende Gewebeschädigung verursacht wird“, wohingegen von chronischem Schmerz gesprochen wird, wenn dieser „dauerhaft oder wiederkehrend für mindestens drei Monate vorhanden ist und die akute Warnfunktion der physiologischen Schmerzwahrnehmung fehlt“ [9]. Pflegerisches Planen und Handeln in der Schmerztherapie orientiert sich dabei an den drei Dimensionen des biopsychosozialen Modells. Im Bereich der palliativen Versorgung orientieren sich Pflegefachpersonen zunehmend am Total-Pain-Modell von Cicely Saunders, das die Spiritualität als eine vierte Dimension einbezieht [5].

In der Versorgung von Patient:innen mit schweren chronischen Schmerzen gilt als Goldstandard die interdisziplinäre multimodale Schmerztherapie, deren zentrales Merkmal die Zusammenarbeit unterschiedlicher Professionen in einem Behandlungsteam ist [28].

Damit pflegetherapeutisches Handeln bestmöglich in die multiprofessionelle Versorgung von Schmerzpatient:innen eingebracht werden kann, ist eine gestufte Qualifizierung der Pflegefachpersonen erforderlich. Seit 2021 liegt von der Deutschen Schmerzgesellschaft ein Programm der Qualifizierung zum pflegerischen Schmerzmanagement vor, das gleichermaßen die berufsfachschulische und die hochschulische Aus‑, Fort- und Weiterbildung von Pflegefachpersonen adressiert. Es umfasst die Stufen 4–7 des Deutschen Qualifikationsrahmens (DQR), also alle Stufen von der berufsfachschulischen dreijährigen Ausbildung bis hin zu einer Masterqualifikation als Advanced Practice Nurse (APN). Im Curriculum wird betont, dass Menschen mit Schmerzen eine Kombination von nichtmedikamentösen und medikamentösen Interventionen benötigen [7].

Das Phänomen Schmerz ist für die pflegerische Versorgung so relevant, dass das Deutsche Netzwerk für Qualitätsentwicklung in der Pflege (DNQP) bereits 2004 den nationalen Expertenstandard „Schmerzmanagement in der Pflege bei akuten Schmerzen“ und 2013 „Schmerzmanagement in der Pflege bei chronischen Schmerzen“ entwickelt und veröffentlicht hat, die 2020 zusammengeführt wurden [9]. Die nationalen Expertenstandards des DNQP sind evidenzbasierte Qualitätsinstrumente, die sich auf komplexe und interaktionsreiche Pflegehandlungen beziehen. Sie gelten als „vorweggenommene Sachverständigengutachten“, die bei juristischen Auseinandersetzungen als Maßstab zur Beurteilung des aktuellen Stands der medizinisch-pflegewissenschaftlichen Erkenntnisse herangezogen werden. Zur Sicherung und Weiterentwicklung in der Pflege nach § 113a Sozialgesetzbuch (SGB) XI ist die Umsetzung der Expertenstandards für zugelassene Pflegeeinrichtungen unmittelbar verbindlich.

Leitlinien zu chronischen Schmerzen betonen die Bedeutung nichtmedikamentöser und edukativer Ansätze

Sowohl im Expertenstandard „Schmerzmanagement in der Pflege“ als auch in nationalen und internationalen Leitlinien zu chronischen Schmerzen wird die Bedeutung nichtmedikamentöser Maßnahmen (beispielsweise Wärme- und Kälteanwendungen) und edukativer Ansätze, wie der Vermittlung von Selbstmanagementstrategien im Umgang mit dem Schmerz, betont. Dennoch ist die medikamentöse Therapie die am häufigsten angewendete Maßnahme bei chronischen Schmerzen [2, 4, 14, 22].

Ziel des vorliegenden Beitrags ist es, einen Einblick in das Potenzial naturheilkundlicher Pflegeinterventionen für die Versorgung von Schmerzpatient:innen zu geben, wobei der Fokus auf sogenannten äußeren Anwendungen wie Wickeln und Auflagen liegt, die risikoarm und einfach anzuwenden sind, sodass sie zur Selbstanwendung geeignet sind. Ältere Schmerzpatient:innen profitieren möglicherweise besonders von ergänzenden oder alternativen Maßnahmen zur medikamentösen Schmerztherapie, da mit zunehmendem Alter das Risiko von Polypharmazie, das heißt der Einnahme von mehr als fünf Medikamenten steigt [10] und dadurch per se bereits eine erhöhte Gefahr von unerwünschten Arzneimittelwirkungen und Interaktionen besteht.

## Definitionen

Zunächst werden relevante Begriffe aus dem Bereich der naturheilkundlichen Pflege definiert.

### Naturheilkundliche Pflegeinterventionen

Naturheilkundliche Pflegeinterventionen werden definiert als pflegerische Interventionen, die im Rahmen von Hydrotherapie, Diätetik, Phytotherapie, Bewegungstherapie und Ordnungstherapie eingesetzt werden. Äußere Anwendungen wie Wickel, Bäder, Auflagen und Einreibungen bilden den Schwerpunkt der naturheilkundlichen Pflegeinterventionen [26]. Eine Überschneidung gibt es zum Begriff der Hausmittel, die als einfache Maßnahmen zur Symptombekämpfung bei leichten gesundheitlichen Beschwerden definiert werden [23]. Der Begriff Hausmittel wird am ehesten dann verwendet, wenn es sich um naturheilkundliche Anwendungen durch Laien und nicht durch Pflegefachpersonen handelt.

### Äußere Anwendungen

Äußere Anwendungen in der Pflege sind nach Fringer et al. [11] therapeutische Interventionen, bei denen gezielt direkte oder indirekte Berührungsarten in Bezug auf das Sinnesorgan Haut und/oder das Nerven-Sinnes-System eingesetzt werden. Ziel ist es, Prozesse anzuregen, die Beschwerden lindern, Erkrankungen heilen und das Wohlbefinden steigern. Äußere Anwendungen wirken „systemisch (mechanisch, physiologisch, psychologisch, spirituell sowie sozial) primär reziprok zwischen Therapeut und dem zu behandelnden Menschen sowie sekundär zwischen seinen Angehörigen und seinem Umfeld“ [11].

Mitte der 1980er-Jahre begann allmählich die Wiederentdeckung der Äußeren Anwendungen

Bis Anfang der 1970er-Jahre waren Äußere Anwendungen fester Bestandteil der Pflegeausbildung und Pflegepraxis. Dann galten sie als veraltet und nicht evidenzbasiert, sodass sie fast vollständig aus dem Alltag der pflegerischen Versorgung verschwanden, außer in der anthroposophisch orientierten Pflege und im Rahmen von Kneipp-Therapien. Mitte der 1980er-Jahre begann allmählich die Wiederentdeckung der dann als „alternative Pflegemethoden“ bezeichneten Maßnahmen. Von den Pflegefachpersonen wurde erkannt, dass sie durch diese Pflegeinterventionen nicht nur Symptome, beispielsweise Schmerzen, lindern, sondern die Patient:innen gleichzeitig in ihrer Selbstpflege und Gesundheitserhaltung, in der Prävention und Gesundheitsförderung unterstützen können [20].

### Aromatherapie

Die Aromatherapie ist ein Teilbereich der Phytotherapie. Während Phytotherapie die Wirkung nichtdestillierter Pflanzenextrakte und Duftstoffe beschreibt, befasst sich die Aromatherapie mit der Anwendung ätherischer Öle und deren physischen, psychosomatischen, psychologischen und physiologischen Wirkungen [25]. Zur nichtmedikamentösen Behandlung des Schmerzes bietet die Aromatherapie zahlreiche Anwendungsmöglichkeiten. In der Pflegepraxis erfolgt die Anwendung hauptsächlich durch die direkte oder indirekte Inhalation oder topisch in einer Mischung mit fettem Pflanzenöl, beispielsweise durch Einreibungen, Streichungen, Waschungen und/oder Einsatz von Wickeln und Auflagen. Ätherische Öle werden zur Linderung akuter und chronischer Schmerzen eingesetzt [21].

### Rhythmische Einreibungen

Die rhythmischen Einreibungen wurden vor dem Hintergrund der anthroposophischen Medizin von den Ärztinnen Ita Wegman und Margarethe Hauschka entwickelt und von Pflegefachpersonen für die professionelle Anwendung und den Pflegealltag modifiziert und konkretisiert. Kennzeichen einer rhythmischen Einreibung ist eine sehr behutsame Berührungsintensität, bei der die Hand des Einreibenden den Körper in rhythmischen, meist kreisenden Streichbewegungen behandelt. Rhythmische Einreibungen werden unter anderem zur Linderung von Schmerzen eingesetzt [13, 16].

## Anwendung naturheilkundlicher Pflegeinterventionen zur Schmerzlinderung in Forschungsprojekten

In der integrativen Medizin liegen inzwischen für komplementäre und naturheilkundliche Verfahren wie Akupunktur, Phytotherapie oder Mind-body-Verfahren zunehmend positive Ergebnisse aus randomisierten, kontrollierten Studien oder Metaanalysen vor. Im Jahr 2021 wurde die S3-Leitlinie zur Komplementärmedizin in der Onkologie veröffentlicht [17]. Für naturheilkundliche Interventionen in der Pflege liegen bisher kaum Studien vor.

Für naturheilkundliche Interventionen in der Pflege liegen bisher kaum Studien vor

Um die Lücke zwischen Expert:innenwissen und externer Evidenz zu verkleinern, wurde von der interprofessionellen Arbeitsgruppe „Integrative Pflege in der Onkologie“ ein Verfahren zur Generierung bestmöglicher Evidenz zu naturheilkundlichen Pflegeinterventionen entwickelt [6]. Das Verfahren hat das Ziel, die pflegerische Expertise in der Anwendung integrativer Pflegemaßnahmen im Bereich der Versorgung onkologischer Patient:innen strukturiert für ausgewählte Pflegephänomene zu sammeln, zu bewerten und evidenzbasierte Handlungsempfehlungen zu formulieren [26]. Es ist auf die Versorgung nichtonkologischer Patient:innen und Patienten und auf Verfahren der naturheilkundlichen Schmerztherapie übertragbar.

Vergleichbar zur beschriebenen Methodologie wurde in den folgenden Forschungsprojekten eine systematische Literaturrecherche mit einer strukturierten Integration von Expert:innenwissen kombiniert. Das Ziel war jeweils die Entwicklung zielgruppenspezifischer naturheilkundlicher Interventionen (beispielsweise zur Linderung von Schmerzen), die im Rahmen der Forschungsprojekte eingesetzt wurden. Zentraler Bestandteil jeder Intervention war die Beratung und Anleitung der Patient:innen und gegebenenfalls ihrer Angehörigen.

### CONGO-Studie (2014–2016)

**Figure 1:**
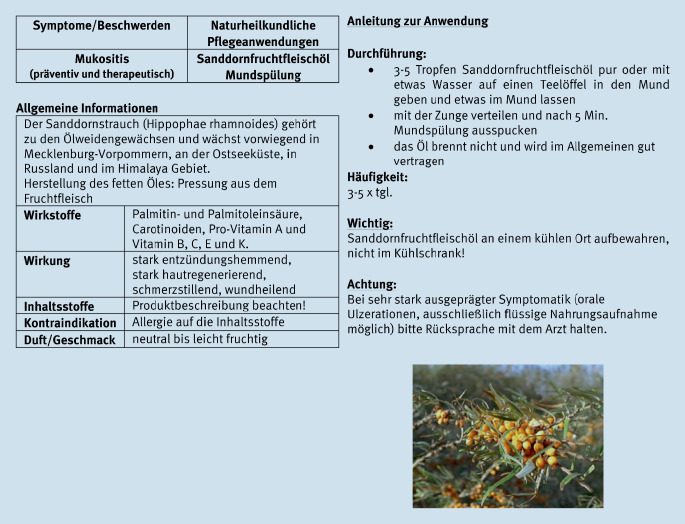

In der vom Bundesministerium für Bildung und Forschung (BMBF) geförderten randomisierten, kontrollierten Studie Complementary Nursing in Gynaecologic Oncology (CONGO) war es das Ziel, den Nutzen und die Wirksamkeit komplementärer Pflege- und Beratungsmaßnahmen bei Patientinnen mit gynäkologischen Tumoren (*n* = 251) während der Chemotherapie zu untersuchen [15, 19]. Für zwölf häufige Beschwerden, die während der Chemotherapie auftreten, wurden 37 äußere Anwendungen von erfahrenen Pflegefachpersonen, Ärzt:innen und einer Psychologin nach dem World-Café-Prinzip ausgewählt und standardisiert. Zur Linderung von Schmerzen und zur Prävention oder Therapie einer Mukositis wurde den Patientinnen als Pflegeintervention eine Mundspülung mit Sanddornfruchtfleischöl empfohlen. Eine Anleitung zur Durchführung der Intervention wurde den Patientinnen nach ausführlicher Beratung und Anleitung mit nach Hause gegeben (Abb. 1a, b).

### HoPES3-Studie (2019–2020)

Im Rahmen der vom BMBF geförderten clusterrandomisierten Studie Holistic Care Program to Integrate Spiritual Needs, Social Activity and Self-Care into Disease Management of Elderly Patients in Primary Care (HoPES3) wurde die Nutzung von Hausmitteln durch ältere Patient:innen (Alter ≥ 70 Jahre, ≥ 3 chronische Erkrankungen, ≥ 3 verordnete Medikamente, Disease-Management-Programm-Teilnahme) untersucht. Ziel war die Verbesserung der hausärztlichen Versorgung älterer, chronisch erkrankter Patient:innen (*n* = 297) durch Stärkung ihrer persönlichen Kraftquellen (spirituelle Bedürfnisse, soziale Kontakte und Selbstfürsorge). Ein Katalog naturheilkundlicher Pflegeinterventionen, die bei häufigen Beschwerden älterer Menschen empfohlen werden können, wurde auf Grundlage einer Literaturrecherche und einer Online-Befragung von Expert:innen für naturheilkundliche Pflegeinterventionen erstellt.

**Figure 2:**
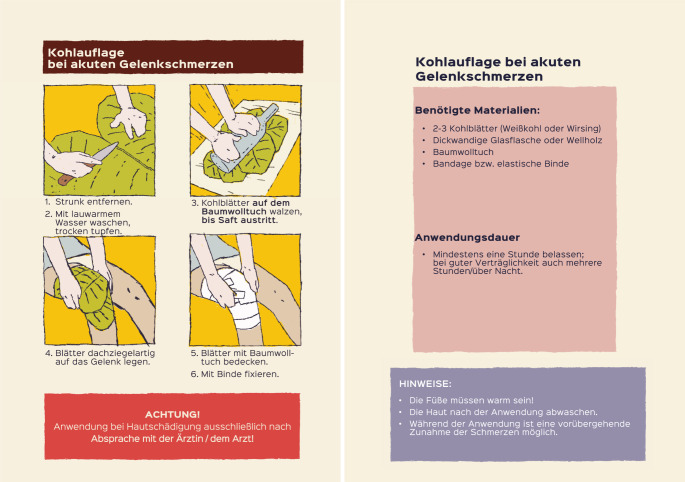

Unter Berücksichtigung der Empfehlungen der „Leitlinie evidenzbasierte Gesundheitsinformation“ [18] wurden schriftliche Anleitungen („Infozepte“) für 17 Hausmittel bei neun Beschwerden entwickelt, unter anderem zu akuten und chronischen Gelenkschmerzen [27]. Ziel der „Infozepte“ war es, die Maßnahmen didaktisch so aufzubereiten und zu beschreiben, dass sie von den Patient:innen selbstständig und korrekt durchgeführt werden können. Die Schritt-für-Schritt-Anleitung, bei der Bilder den Text veranschaulichen, erleichtert eine sichere Anwendung zu Hause. Bei akuten und chronischen Gelenkbeschwerden wurde die Kohlauflage empfohlen ([3, 13, 24]; Abb. 2).

### Das Projekt CCC-Integrativ (2019–2022)

**Figure 3:**
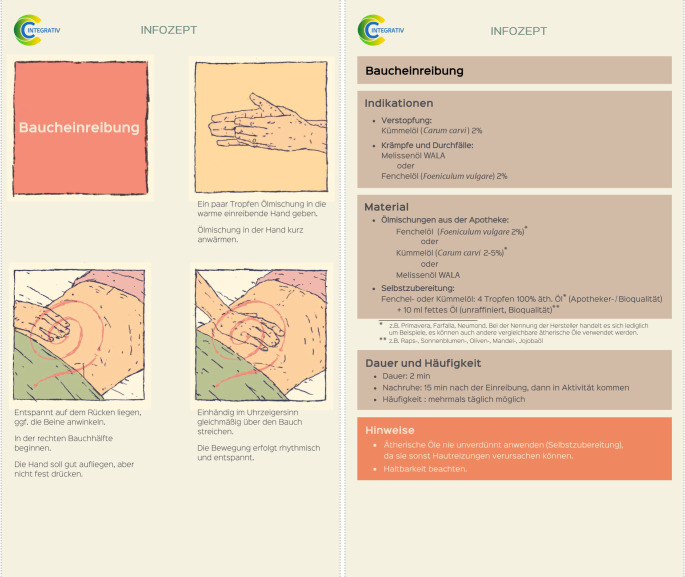

Im vom Innovationsfonds geförderten Projekt CCC-Integrativ (Implementierung eines sektorenübergreifenden, interprofessionellen Programms zur evidenzbasierten Beratung von Krebspatient:innen im Bereich Komplementäre Medizin und Pflege [KMP] an Comprehensive Cancer Centers [CCCs] in Baden-Württemberg) werden Patient:innen individuell zu Chancen und Risiken von KMP beraten („empowert“), sodass sie selbstständig entscheiden können, ob sie KMP in Anspruch nehmen wollen und wenn ja welche [29]. Die Beratungen wurden von interprofessionellen Teams aus im Projekt geschulten Ärztinnen und Ärzten sowie Pflegenden angeboten. Zu 19 häufigen Beschwerden von Krebspatient:innen, beispielsweise Mukositis, chemotherapieinduzierter Polyneuropathie, Schlafstörung und Diarrhö, wurden in einem strukturierten Konsensusverfahren 44 naturheilkundliche Pflegeinterventionen ausgewählt, die zur Selbstanwendung geeignet sind. Bei schmerzhaften Bauchkrämpfen im Rahmen einer Diarrhö wurde eine Baucheinreibung mit einer Melissenölmischung empfohlen, die bei Bedarf anhand eines „Infozepts“ zur Selbstdurchführung angeleitet wurde. Die Melissenölmischung enthält ätherische Öle aus Fenchel (*Foeniculum vulgare*), Melisse (*Melissa officinalis*), Kümmel (*Carum carvi*) und Majoran (*Origanum majorana*; [3, 13, 25]). Die konkrete Anleitung zur Baucheinreibung bei schmerzhaften Bauchkrämpfen ist in Abb. 3 dargestellt.

## Weitere naturheilkundliche Pflegeinterventionen zur Schmerzlinderung

In Tab. 1, 2, 3, 4 und 5 wird ein Überblick über das breite Spektrum an Indikationen und Maßnahmen naturheilkundlicher Pflegeinterventionen bei unterschiedlichen akuten und chronischen Schmerzen gegeben [3, 13, 24]. Viele naturheilkundliche Substanzen lassen sich auch mittels einfacher Auftragung oder Einreibungen anwenden. Die Einreibung wird sanft, ohne oder mit wenig Druck ausgeführt. Die Anwendungsarten sind Auflagen und Einreibungen mit ätherischen Ölen, Essenzauflagen, feucht-kalte und feucht-warme Auflagen sowie hautreizende Substanzen.

### Auflagen oder Einreibungen mit ätherischen Ölen zur Schmerzlinderung

Zur Anwendung (Tab. 1) kommen 100 % naturreine ätherische Öle in Apotheker- oder Bioqualität, die mit fetten Ölen gemischt werden. Die Konzentration beträgt 0,5–10 %.

**Table 1:** 

Anwendung	Indikation
WALA Aconit Schmerzöl	Gelenkschmerzen aus dem rheumatischen Formenkreis, Herpes-zoster-Schmerzen, Nervenschmerzen
Weleda Arnica comp. Formica (ölige Einreibung)	Polyneuropathien (auch chemotherapieinduziert)
Echte Kamille (*Matricaria-chamomilla*-Öl)	Bauchkrämpfe
WALA Melissenöl (ölige Einreibung)	Bauchkrämpfe unter Diarrhö
Lavendel fein (Ölauflage)	Schmerz
WALA Solum Öl (ölige Einreibung)	Muskelschmerzen, Verspannungen
Pfefferminzöl (*Mentha piperita*)	Spannungskopfschmerz

### Essenzauflagen zur Schmerzlinderung

Essenzen sind ein alkoholischer Pflanzenauszug. Für eine Essenzauflage wird eine übliche Dosierung von 150 ml Wasser auf 1 Teelöffel Essenz verwendet. Die Anwendung kann mehrmals über den Tag bei unterschiedlichen Indikationen verteilt wiederholt werden (Tab. 2).

**Table 2:** 

Anwendung	Indikation
*Arnica*-Essenz 20 %	Verletzungen/Verstauchungen Rheumatoide Gelenkerkrankungen
*Calendula*-Essenz 10 %	Entzündete nicht offene Haut Phlebitis
*Oxalis*-Essenz 20 %	Obstipation Menstruationsbeschwerden

### Feucht-kühle Anwendungen zur Schmerzlinderung

Die Temperatur einer kühlen Anwendung beträgt 1–2 °C unter Körpertemperatur. Je nach Empfinden der Patient:innen kann die Temperatur auch niedriger sein. Im Fieberanstieg und bei kalten Extremitäten dürfen diese Maßnahmen nicht angewendet werden. Mögliche Anwendungen sind in Tab. 3 aufgeführt.

**Table 3:** 

Anwendung	Indikation
Quarkauflage	Schmerzen bei einer aktivierten Arthrose
Prießnitz-Wickel	Schmerzen bei einer akuten Halsentzündung

### Feucht-warme und feucht-heiße Anwendungen zur Schmerzlinderung

Feucht-warme bzw. -heiße Anwendungen müssen sehr achtsam durchgeführt werden. Bei fachgerechter Anwendung besteht keine Verbrennungsgefahr für die Patienten. Mögliche Indikationen sind in Tab. 4 aufgeführt.

**Table 4:** 

Anwendung	Indikation
Bauchwickel mit Kamillentee	Krampfartige Bauchbeschwerden
Bauchwickel mit Melissentee	Krampfartige Beschwerden, z. B. bei Diarrhö
Bauchwickel oder Leberwickel mit Schafgarbentee	Menstruationsbeschwerden Leberkapselschmerz (Metastasen)
Feucht-heißer Gelenkwickel	Chronische Gelenkschmerzen
Leinsamenauflage (Kataplasma)	Knochenschmerzen Gelenkbeschwerden Gerstenkorn Nagelbettentzündung Sinusitis

### Anwendungen mit hautreizenden Substanzen zur Schmerzlinderung

Hautreizende Substanzen (Tab. 5) reizen die Haut meist schon nach wenigen Minuten. Dosierung und Anwendung sind abhängig von Qualität und Frische der Substanz. Eine Ausnahme bilden Zwiebeln. Sie können längere Zeit aufgelegt werden.

**Table 5:** 

Anwendung	Indikation
Zwiebelauflage	Otitis media
Meerrettich frisch gerieben	Nackenverspannungen

## Diskussion und Schlussfolgerung

Dieser Beitrag zeigt, dass es inzwischen eine Vielzahl an nichtmedikamentösen naturheilkundlichen Anwendungen zur Schmerzlinderung gibt, die in einem systematischen Konsensusverfahren von Pflegeexpert:innen entwickelt wurden und die von geschulten Pflegefachpersonen selbständig angewendet werden können. Relevant ist in diesem Zusammenhang natürlich, dass – wie im Expertenstandard „Schmerzmanagement in der Pflege“ dargelegt – eine interprofessionelle Zusammenarbeit bei der Durchführung der multimodalen Schmerztherapie sowie ein guter Austausch und eine gute Kommunikation der beteiligten Professionen erfolgen. Von Bedeutung ist, dass gegenseitiges Vertrauen besteht und die jeweiligen Kompetenzen der beteiligten Professionen bekannt sind. Es hat sich gezeigt, dass sich eine respektvolle Zusammenarbeit gemeinsam mit den Erkrankten, Angehörigen, Begleitern und Teammitgliedern positiv auf die Patientenoutcomes, beispielsweise auf die Schmerzlinderung, auswirkt [9].

Naturheilkundliche Maßnahmen können die Selbstwirksamkeit der Patient:innen stärken

Relevant ist weiter eine fundierte Schulung der Pflegefachpersonen in der Anwendung naturheilkundlicher Pflegeinterventionen zur Schmerzlinderung. Interprofessionelle Fort- und Weiterbildungen können hier einen wichtigen Beitrag leisten, indem das Verständnis füreinander gestärkt wird und gegenseitige Rollen und Kompetenzen in der Versorgung geklärt werden.

Die Qualifikation zur Pain Nurse oder algesiologischen Fachassistenz umfasst auch nichtmedikamentöse Pflegeinterventionen, zu denen die naturheilkundlichen Interventionen zählen. Zukünftig ist vorstellbar, dass diese Weiterqualifikation auch in Masterstudiengängen eingebunden wird und zur Advanced Practice Nurse qualifiziert [12]. Hier ist neben der Vermittlung evidenzbasierter Pflege auch die Qualifikation zu Verfahren der Schmerzlinderung wie der Akupunktur möglich, die aktuell nur von Ärzt:innen oder Heilpraktiker:innen durchgeführt werden können. So kann ein weiteres Handlungsfeld für akademisierte Pflegefachpersonen erschlossen werden. Schon jetzt ist die Durchführung von Akupressur für Pflegefachpersonen mit entsprechender Schulung möglich [8].

Patient:innen, insbesondere mit chronischen Schmerzen, wünschen sich häufig eine „sanftere“ Therapie, um sowohl die Wechsel- und Nebenwirkungen der Schmerzmedikation zu vermeiden als auch das Gefühl zu haben, „selbst etwas tun zu können“. Naturheilkundliche Maßnahmen leisten hier einen wesentlichen Beitrag, da sie von den Patient:innen selbst durchgeführt werden können und so die Selbstwirksamkeit der Patient:innen gestärkt wird [1].

Diese Möglichkeit der Erweiterung eines autonomen Handlungsfelds in der Anwendung nichtmedikamentöser Verfahren wie auch die Beratung und Schulung von Patient:innen in den Anwendung machen den Pflegeberuf attraktiver, da ein eigenverantwortliches Handeln gefördert wird.

## Fazit für die Praxis

- In diesem Beitrag wurde gezeigt, dass es eine Vielzahl nichtmedikamentöser, naturheilkundlicher Pflegeinterventionen zur Schmerzlinderung gibt, insbesondere Äußere Anwendungen wie Wickel, Auflagen und Einreibungen.
- Um die Lücke zwischen Expert:innenwissen und externer Evidenz zu verkleinern, wurde ein systematisches Konsensusverfahren zur Generierung bestmöglicher Evidenz zu naturheilkundlichen Pflegeinterventionen entwickelt.
- Nach Beratung und Anleitung durch Pflegefachpersonen können risikoarme und einfache Anwendungen von den Patient:innen selbst durchgeführt werden.
- Die Befähigung zur Selbstfürsorge kann die Selbstwirksamkeit der Patient:innen stärken.
- Die Anwendung nichtmedikamentöser, naturheilkundlicher Verfahren wie auch die Beratung und Schulung von Patient:innen in den Anwendung machen den Pflegeberuf attraktiver, da ein eigenverantwortliches Handeln gefördert wird.
